# Dynamic Edge Loading Balancing with Edge Node Activity Prediction and Accelerating the Model Convergence

**DOI:** 10.3390/s25051491

**Published:** 2025-02-28

**Authors:** Wen Chen, Sibin Liu, Yuxiao Yang, Wenjing Hu, Jinming Yu

**Affiliations:** School of Information Science and Technology, Donghua University, Shanghai 201620, China; chenwen@dhu.edu.cn (W.C.); 2222134@mail.dhu.edu.cn (Y.Y.); 2222137@mail.dhu.edu.cn (W.H.); yujinming@dhu.edu.cn (J.Y.)

**Keywords:** deep reinforcement learning, load balancing, mobile edge computing, resource allocation, task offloading

## Abstract

In mobile edge computing networks, achieving effective load balancing across edge server nodes is essential for minimizing task processing latency. However, the lack of a priori knowledge regarding the current load state of edge nodes for user devices presents a significant challenge in multi-user, multi-edge node scenarios. This challenge is exacerbated by the inherent dynamics and uncertainty of edge node load variations. To tackle these issues, we propose a deep reinforcement learning-based approach for task offloading and resource allocation, aiming to balance the load on edge nodes while reducing the long-term average cost. Specifically, we decompose the optimization problem into two subproblems, task offloading and resource allocation. The Karush–Kuhn–Tucker (KKT) conditions are employed to derive the optimal strategy for communication bandwidth and computational resource allocation for edge nodes. We utilize Long Short-Term Memory (LSTM) networks to forecast the real-time activity of edge nodes. Additionally, we integrate deep compression techniques to expedite model convergence, facilitating faster execution on user devices. Our simulation results demonstrate that our proposed scheme achieves a 47% reduction in terms of the task drop rate, a 14% decrease in the total system cost, and a 7.6% improvement in the runtime compared to the baseline schemes.

## 1. Introduction

With the advent of fifth-generation mobile communication technology, there has been an exponential growth in the number of smart devices, giving rise to a plethora of resource-intensive and latency-sensitive applications. These include online video streaming, online education, telemedicine, augmented reality, etc. [1]. Achieving satisfactory Quality of Service (QoS) and Quality of Experience (QoE) for such applications is challenging when relying on the limited computing capabilities of mobile devices alone [2].

Traditional approaches often depend on centralized cloud computing infrastructures, where data processing and storage tasks are managed in distant data centers [3]. However, this method necessitates extensive data transmission between users and data centers, potentially leading to prohibitively high latency. In response, mobile edge computing (MEC) has risen as a compelling alternative, attracting considerable interest from both industry and academia [4].

MEC addresses these challenges by relocating computation and storage resources to the network’s edge, closer to the users. This relocation facilitates real-time processing and significantly reduces latency, making MEC an attractive option for applications that are sensitive to latency [5].

By situating computing resources in close proximity to the end-users, such as at base stations or routers, MEC enables localized data processing. This effectively eliminates the need for data to be sent back to remote data centers [6,7]. This strategy not only enhances the responsiveness of applications but also mitigates network congestion. This is particularly beneficial in scenarios with high traffic volumes and stringent low-latency requirements, such as intelligent transportation systems [8].

In the MEC architecture, edge nodes are tasked with both computation and storage responsibilities. However, in real-world scenarios characterized by unpredictable task arrivals, a sudden surge of tasks offloaded to a single edge node can lead to overloading and prolonged operation over time. Unlike cloud service centers, edge nodes in practical deployments typically have limited computing and storage resources [9]. Consequently, tasks processed on overloaded edge nodes may experience extended queuing delays, potentially resulting in untimely processing or even task abandonment.

To maintain load balancing across edge nodes, it is crucial to establish reasonable and effective task offloading policies. Additionally, when tasks are transmitted to edge nodes via wireless channels, the allocation of communication bandwidth and computational resources becomes pivotal to the overall system performance [10]. Poor resource allocation can lead to high latency and suboptimal resource utilization, which are clearly undesirable. Therefore, the optimization of MEC systems necessitates the design of an effective resource allocation strategy.

Several studies have addressed load balancing across edge nodes in the context of MEC task offloading. In [11], the task offloading problem in a dynamic MEC environment with rapidly moving user devices is considered. The authors use Lyapunov optimization theory and a neural network framework to balance the load across different cloudlets. In [12], to address the computational load imbalance problem on edge nodes, the authors employ a D3QN-based deep reinforcement learning framework to group MEC nodes. The work in [13] proposes a heuristic offloading algorithm to balance computational and traffic load. However, these studies overlook the queuing delays that occur during task offloading. The study in [14] investigates delay estimation and computational task offloading in MEC networks for V2X applications, developing an end-to-end delay prediction framework that integrates various delays using actual round-trip time data. In [15], the authors address the offloading problem in MEC networks with inter-task dependencies by assigning tasks to different waiting queues based on their priority and deadline requirements. While these studies consider queuing delays associated with task offloading, they do not focus on the load balancing problem in dynamic scenarios, neglecting the load state information of each edge node during offloading decisions.

Building on these considerations, this paper proposes a distributed task offloading and resource allocation strategy leveraging deep reinforcement learning to tackle the challenge posed by the dynamic uncertainty of edge node loads in multi-user, multi-edge node environments. The primary objective is to minimize the long-term average cost of the user system in terms of latency and energy consumption. Specifically, each user device predicts the activity state of edge nodes in real time by leveraging historical load information, enabling adaptive offloading decisions. Subsequently, the optimal allocation of communication bandwidth and computational resources is determined to maximize system resource utilization. Furthermore, to reduce the computational complexity and enhance the execution speed of the deep learning model, we incorporate deep compression techniques [16] within the algorithm. These techniques, including pruning and quantization, effectively reduce the model’s storage requirements and computational costs, making them suitable for deployment in resource-constrained environments. Finally, simulation results demonstrate that the proposed scheme outperforms the baseline approaches in reducing the task drop rate, lowering the long-term average cost of the user’s system, and minimizing runtime.

In summary, the primary contributions of this paper are as follows:

- We explore the problem of dynamic task offloading and resource allocation in a multi-user, multi-edge node scenario, considering the uncertainty of edge node load variations in dynamic environments, with the objective of minimizing the long-term average cost of the user system in terms of latency and energy consumption.
- We propose a solution for real-time task offloading and resource allocation. We decompose the optimization problem into two subproblems, task offloading and resource allocation. Using the Karush–Kuhn–Tucker (KKT) conditions, we derive the optimal communication bandwidth and computational resource allocation scheme for edge nodes. We then propose the Balanced Preserve based on Deep Reinforcement Learning (BPDRL) algorithm, which enables each user device to dynamically predict the load of the edge nodes based on historical information and independently make offloading decisions without knowing the load of other devices, achieving load balancing at the edge nodes.
- To expedite model convergence and facilitate efficient deployment on user devices, we incorporate deep compression techniques into the algorithm to reduce model size. Simulation experiments validate the feasibility and effectiveness of the proposed scheme.

The rest of this paper is organized as follows. We review related work in Section 2. In Section 3, we introduce the system model. The corresponding optimization problem is established in Section 4. The BPDRL algorithm is illustrated in Section 5. In Section 6, we give the performance evaluation. Finally, Section 7 summarizes the work of this paper.

## 2. Related Works

In MEC, the implementation of effective task offloading and resource allocation strategies is crucial for enhancing system efficiency and delivering a satisfactory service experience to users. Consequently, this area has remained a central and consistently emphasized focus within the research community.

A multitude of studies have been conducted on the challenges of computation offloading and resource allocation in MEC environments. For instance, Wu et al. [10] propose a game-theoretic approach to multi-cellular network computing offloading, which effectively reduces the system latency and energy consumption. Peng et al. [17] investigate the problem of task offloading and resource allocation in MEC-enabled dynamic networks, utilizing an actor–critic deep learning algorithm to minimize the long-term average tasks completion delay. Zhang et al. [18] consider two task offloading modes and propose a greedy-based edge node selection strategy to reduce overall task execution delay. Li et al. [19] formulate the computation offloading and resource optimization as a Mixed Integer Nonlinear Programming (MINLP) problem, employing genetic algorithms and Monte Carlo search methods to jointly optimize offloading strategies and bandwidth resources. Zeng et al. [20] maximize the system QoE by jointly optimizing task offloading strategies and multi-dimensional resource allocation. Xia et al. [21] propose a vehicle location-aware task offloading mechanism, differentiating between single-unit and multi-unit offloading scenarios. Chen et al. [22] study multi-user computation offloading in a dynamic MEC environment with energy harvesting capabilities, designing a dynamic offloading algorithm to minimize system energy consumption under delay constraints. Chen et al. [23] model the offloading problem as a Markov decision process and address the state space explosion issue using the Deep Deterministic Policy Gradient (DDPG) algorithm.

However, the aforementioned studies overlook the load status of edge nodes during task offloading. Real network environments are inherently dynamic and uncertain, leading to significant unpredictability in the real-time load of edge nodes. Given the limited computation resources of edge nodes, managing all incoming tasks becomes a complex challenge. Zhu et al. [24] design an immune algorithm-based task offloading scheme aimed at optimizing system task response time, device energy consumption, and server load balance to ensure system service quality and load balancing performance. Yet, this approach does not account for task offloading over continuous time intervals, making it less suitable for dynamic environments. Yan et al. [25] focus on the task offloading problem for unmanned rescue vehicles, aiming to minimize task drop rates, system delay, and energy consumption. They achieve edge server load balancing by equalizing the number of queued tasks. However, this research is conducted in a quasi-static environment and may not be applicable to truly dynamic settings. Tang et al. [26] design a distributed offloading algorithm that addresses the dynamic uncertainty of edge node loads by integrating Long Short-Term Memory (LSTM) networks, Deep Dueling Q Networks, and Double Deep Q-Network (DDQN) techniques. Chen et al. [27] explore the computation offloading problem in ultra-dense software-defined networks, proposing a load balancing algorithm based on load estimation to mitigate the ping-pong effect. These studies simplify the communication model of wireless networks and do not consider the impact of communication resource constraints on the network. While recent studies [12,28,29] have primarily focused on task offloading and load balancing in mobile edge computing, our research emphasizes dynamic task offloading and resource allocation to address the challenges posed by dynamic variations in edge node load, distinguishing it from the aforementioned works.

Deep reinforcement learning (DRL), a prominent subfield of machine learning (ML), has emerged as a potent approach for tackling computation offloading and resource allocation challenges in MEC. This approach integrates deep learning with reinforcement learning, enabling the system to intelligently determine computation task offloading and resource allocation strategies. Wu et al. [30] categorize users into regular and VIP users, proposing a DRL-based task offloading algorithm to adapt the fluctuating task arrival rates in MEC networks. Cao et al. [31] employ fuzzy inference to assess network traffic patterns and propose a reinforcement learning-based algorithm to devise offloading strategies. Zhao et al. [32] present a DRL-based offloading strategy to prevent suboptimal decisions by minimizing state-space dimensions while considering task data dependencies and vehicle mobility. Hu et al. [33] optimize the distribution of system energy and computational resources in a dynamic single MEC environment by refining exploration and experience replay components within a DRL-based algorithm. However, DRL models often require considerable computational resources for both training and execution, posing issues such as high computational complexity, energy consumption, and reduced deployment efficiency on user devices. To counter these challenges, we integrate deep compression techniques into our DRL model. This integration significantly reduces model size and computational complexity, thereby improving deployment efficiency through strategies like pruning and parameter quantization. Table 1 contrasts the salient features and unique aspects of our research against relevant existing studies.

## 3. System Model

**Figure 1:**
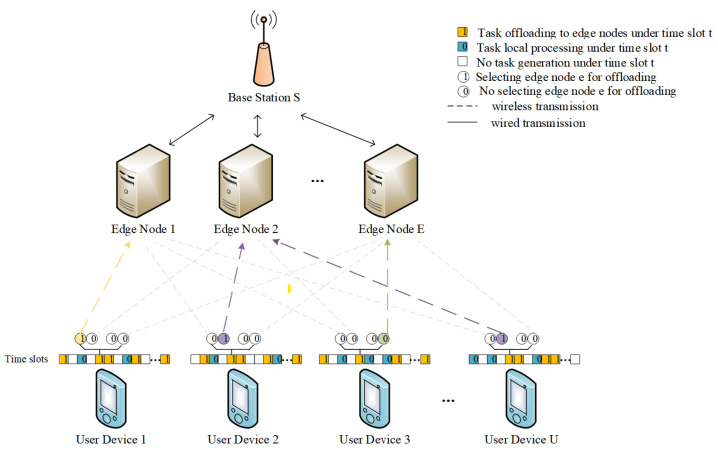
System model.

In this paper, we consider a MEC system containing a base station *S*, a set of edge nodes E={1,2,…,E}, and a set of user devices U={1,2,…,U}. The base station is equipped with multiple MEC nodes, and user devices make decisions on whether to execute tasks locally or delegate them to the MEC nodes affiliated with the base station, in accordance with predefined task offloading strategies as illustrated in Figure 1. It is presumed that users within the base station’s communication radius access the network via Orthogonal Frequency Division Multiple Access (OFDMA). Moreover, the wireless communication between user devices and the base station is susceptible to path loss and distance-dependent large-scale fading [10]. An in-depth exploration of the communication model is presented in the subsection titled “Communication Model”.

To capture the system’s dynamic characteristics more effectively, we concentrate on the system’s functioning across a series of time slots T={1,2,…,T}, with each time slot lasting a duration of Δ. Within each time slot, each user device has a probabilistic likelihood of generating a random IoT application task, which requires a decision on whether to process it locally or offload it to a designated MEC node. We adopt a triplet $\left(d_{u}\left(t\right),c_{u}\left(t\right),T^{max}\right)$ to represent the task $Task_{u}\left(t\right)$ generated by user device *u* at time slot *t*, where $d_{u}\left(t\right)$ represents the task size in bits, $c_{u}\left(t\right)$ denotes the task processing density, i.e., the CPU cycles required to process each bit of data, and $T^{max}$ indicates the maximum acceptable latency for the task. The task is discarded if the processing duration surpasses this threshold. Additionally, we assume that tasks are non-divisible, meaning that each task must either be processed locally or entirely offloaded to an edge node.

We represent the offloading decision for a task arriving at time slot *t* as $x_{u}\left(t\right)\in \left\{0,1\right\}$, where $x_{u}\left(t\right)=0$ indicates local task processing and $x_{u}\left(t\right)=1$ indicates offloading the task to an edge node for processing via wireless channel transmission. When a task is selected for offloading to an edge node, let $y_{u,e}\left(t\right)$ represent whether the task generated by user device *u* is allocated for processing at edge node *e* during time slot *t*. Specifically, $y_{u,e}\left(t\right)\in \left\{0,1\right\}$, where $y_{u,e}\left(t\right)=1$ confirms that the task is processed at edge node *e* and $y_{u,e}\left(t\right)=0$ confirms that it is not. The variable $y_{u,e}\left(t\right)$ satisfies the following relation:

(1) $$x_{u}\left(t\right)=1⟶\sum_{e\in E}y_{u,e}\left(t\right)=1,u\in U,t\in T$$

This equation indicates that a single edge node must be selected for task offloading.

A summary of the key symbols utilized in this paper is provided in Table 2. In the following, we specify the system in terms of the communication model, the computational model, and the queuing model.

### 3.1. Communication Model

In the wireless communication process between user device *u* and base station *S*, the uplink transmission rate is represented as follows:

(2) $$r_{u}\left(t\right)=w_{u}log_{2}\left(1+\frac{p_{u}g_{u,S}}{N_{0}}\right)$$

where $w_{u}$ denotes the bandwidth allocated to user device *u* by the MEC system, $p_{u}$ denotes the transmit power of user device *u*, $g_{u,S}$ denotes the channel gain between user device *u* and base station *S*, and $N_{0}$ denotes the channel noise power.

When user device *u* offloads $Task_{u}\left(t\right)$ to edge node *e* for processing, the transmission delay incurred through the wireless channel transmission is

(3) $$T_{u}^{trans}\left(t\right)=\sum_{e\in E}\frac{y_{u,e}\left(t\right)d_{u}\left(t\right)}{r_{u}\left(t\right)}$$

The energy consumption generated by user device *u* during the transmission process is

(4) $$E_{u}^{trans}\left(t\right)=p_{u}T_{u}^{trans}\left(t\right)$$

In comparison to the uploaded data, the downlink transmission of output data back to the user device is considered negligible, and thus, the downlink transmission delay is disregarded [34].

### 3.2. Computational Model

#### 3.2.1. Local Computation

When user device *u* opts to execute the task locally, the computational time required is expressed as

(5) $$T_{u}^{local}\left(t\right)=\frac{d_{u}\left(t\right)c_{u}\left(t\right)}{F_{u}^{local}}$$

where $F_{u}^{local}$ represents the local computing capability of user device *u*, i.e., the CPU cycles per second.

The energy consumption generated by the user device *u* during the local computation is represented as [35]

(6) $$E_{u}^{local}\left(t\right)=\kappa T_{u}^{local}\left(t\right){\left(F_{u}^{local}\right)}^{3}$$

where κ is the effective capacitance factor, which is related to the hardware architecture of the user device [36].

#### 3.2.2. Edge Computation

The time required for edge node *e* to process $Task_{u}\left(t\right)$ is

(7) $$T_{u}^{edge}\left(t\right)=\sum_{e\in E}\frac{y_{u,e}\left(t\right)d_{u}\left(t\right)c_{u}\left(t\right)}{f_{e}^{edge}\left(t\right)}$$

where $f_{e}^{edge}\left(t\right)$ denotes the computational resources allocated to edge node *e* in time slot *t*.

Our focus is on the energy consumption at the user device due to computation. Therefore, we omit the energy consumption by the user device when a task is uploaded to the edge server for processing, as it is typically relatively insignificant.

### 3.3. Queue Model

Each user device and edge node is equipped with dedicated task queues that operate on a first-in–first-out basis. Specifically, each user device possesses two separate queues, the computation queue and the transmission queue. In parallel, each edge node manages *U* task queues, where *U* represents the number of user devices associated with that edge node.

**Figure 2:**
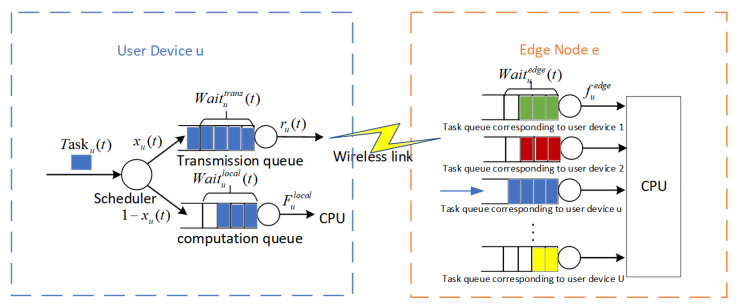
Queuing model.

Prior to the conclusion of time slot *t*, user device *u* generates a new task $Task_{u}\left(t\right)$. This task undergoes evaluation by the scheduler, which ascertains the offloading decision, and is then positioned into the appropriate queue at the commencement of the ensuing time slot. If $Task_{u}\left(t\right)$ is selected for local processing, it is slotted into the computation queue of user device *u* for execution. On the other hand, if the task is destined for offloading to edge node *e*, it is initially placed in the transmission queue of user device *u* before being relayed to the corresponding queue on edge node *e* for additional queuing and subsequent processing through the wireless link. This process is illustrated in Figure 2. For the sake of simplicity, we make $Task_{u}\left(t\right)=0$ when no task is generated during time slot *t*.

#### 3.3.1. Local Queue

(1) Local computational queue.

If $x_{u}\left(t\right)=0$, i.e., $Task_{u}\left(t\right)$ arrives at the local computation queue during time slot *t*, the number of time slots required for $Task_{u}\left(t\right)$ to be processed from the start to the completion, according to the local computation model, is given by

(8) $$Num_{u}^{local}\left(t\right)=\frac{T_{u}^{local}\left(t\right)}{\Delta }$$

Before time *t* when $Task_{u}\left(t\right)$ arrives, if there are pending tasks in the local computation queue that have not been completed, $Task_{u}\left(t\right)$ will not be processed immediately. Instead, it has to wait until all preceding tasks in the computation queue are finished. We assume that the time slot in which $Task_{u}\left(t\right)$ is fully processed in the local computation queue is $Time_{u}^{local}\left(t\right)$; thus, we have the following equation:

(9) $$\begin{array}{c} \begin{array}{c} Time_{u}^{local}\left(t\right)=\min\{t+Num_{u}^{local}\left(t\right)+Wait_{u}^{local}\left(t\right),t+T^{max}\} \end{array} \end{array}$$

where $Wait_{u}^{local}\left(t\right)$ represents the waiting time slots in the local computation queue before $Task_{u}\left(t\right)$ begins processing. The waiting time slots are calculated as

(10) $$Wait_{u}^{local}\left(t\right)=\max\left\{\max_{t^{\prime }\in \left\{0,1,...,t-1\right\}}Time_{u}^{local}\left(t^{\prime }\right)-t,0\right\}$$

Note that if the sum of the waiting time slots $Wait_{u}^{local}\left(t\right)$ and the processing time slots $Num_{u}^{local}\left(t\right)$ exceeds the maximum tolerable time slots, the task will be discarded.

(2) Local transmission queue.

According to the transmission model, if $x_{u}\left(t\right)=1$, i.e., $Task_{u}\left(t\right)$ arrives at the local transmission queue in time slot *t*, the number of time slots required for $Task_{u}\left(t\right)$ to be transmitted from the start of transmission until it is completely sent to the designated edge node is

(11) $$Num_{u}^{trans}\left(t\right)=\frac{T_{u}^{trans}\left(t\right)}{\Delta }$$

Before the arrival of $Task_{u}\left(t\right)$ at time *t*, if there are still tasks in the local transmission queue that have not been transmitted, we assume the time slot when $Task_{u}\left(t\right)$ is fully transmitted in the local transmission queue is $Time_{u}^{trans}\left(t\right)$. Thus, we have

(12) $$\begin{array}{c} \begin{array}{cc} Time_{u}^{trans}\left(t\right)= & \min\{t+Num_{u}^{trans}\left(t\right)+Wait_{u}^{trans}\left(t\right),t+T^{max}\} \end{array} \end{array}$$

where $Wait_{u}^{trans}\left(t\right)$ represents the waiting time slots in the local transmission queue before $Task_{u}\left(t\right)$ begins transmission. The waiting time slots for transmission are determined by

(13) $$Wait_{u}^{trans}\left(t\right)=\max\left\{\max_{t^{\prime }\in \left\{0,1,...,t-1\right\}}Time_{u}^{trans}\left(t^{\prime }\right)-t,0\right\}$$

Similarly, if the sum of the waiting time slots $Num_{u}^{trans}\left(t\right)$ and the transmission time slots $Wait_{u}^{trans}\left(t\right)$ surpasses the task’s maximum tolerable time slots, $Task_{u}\left(t\right)$ will be discarded.

#### 3.3.2. Edge Queue

During time slot *t*, if there is a task arriving at queue *u* of the edge node *e*, the number of time slots required for the task to be processed from the start until completion on edge node *e*, according to the edge computing model, is

(14) $$Num_{u}^{edge}\left(t\right)=\frac{T_{u}^{edge}\left(t\right)}{\Delta }$$

We denote the time slot as $Time_{u}^{edge}\left(t\right)$ when the task is completely processed in edge queue *u*. Thus, we have

(15) $$\begin{array}{c} \begin{array}{cc} Time_{u}^{edge}\left(t\right)= & \min\{t+Num_{u}^{edge}\left(t\right)+Wait_{u}^{edge}\left(t\right),t+T^{max}\} \end{array} \end{array}$$

where $Wait_{u}^{edge}\left(t\right)$ represents the waiting time slots for the task in the edge queue *u*. The waiting time in the edge queue is given by

(16) $$Wait_{u}^{edge}\left(t\right)=\max\left\{\max_{t^{\prime }\in \left\{0,1,...,t-1\right\}}Time_{u}^{edge}\left(t^{\prime }\right)-t,0\right\}$$

Here, we define the active queues $Q_{e}\left(t\right)$ as the set of queues on an edge node *e* that contain tasks which are either being processed or are waiting to be processed, i.e.,

(17) $$Q_{e}\left(t\right)=\left\{u|Wait_{u}^{edge}\left(t\right)>0\;\;or\;\;Task\left(t\right)>0,\;u\in U\right\}$$

The number of active queues on edge node *e* is represented as $|Q_{e}\left(t\right)|$, and assuming that the edge node employs a resource sharing model [26], the real-time computational resources of edge node *e* can be articulated as

(18) $$f_{e}^{edge}\left(t\right)=\frac{F_{e}^{edge}}{|Q_{e}\left(t\right)|}$$

where $F_{e}^{edge}$ denotes the aggregate computational resources available on edge node *e*.

The active queues $Q_{e}\left(t\right)$ on the edge node *e* fluctuate dynamically in real time. When making offloading decisions, user devices cannot preemptively discern the active queues $Q_{e}\left(t\right)$ on an edge node *e*. If multiple user devices concurrently offload tasks to a specific edge node *e*, the node might encounter a scarcity of computational resources. This situation could precipitate an increase in the waiting time slots $Wait_{u}^{edge}\left(t\right)$ for tasks in the queue and the processing time slots $Time_{u}^{edge}\left(t\right)$, potentially causing the sum of these time slots to surpass the maximum tolerable time slots for tasks. Consequently, this could lead to a higher rate of task abandonment. In contrast, when fewer tasks are offloaded to edge nodes, their resource utilization decreases. Therefore, it is essential to develop appropriate offloading and resource allocation strategies to balance the load on edge nodes and fully utilize system resources.

## 4. Problem Formulation

Drawing from the preceding discourse, the cumulative delay encountered by user device *u* throughout the time period *T* can be defined as

(19) $$\begin{array}{c} Time_{u}^{total}=\lim_{T\to \infty }\frac{1}{T}\sum_{t=1}^{T}[\left(1-x_{u}\left(t\right))(Time_{u}^{local}\left(t\right)-t\right)\\ +x_{u}\left(t\right)(Time_{u}^{trans}\left(t\right)+Time_{u}^{edge}\left(t\right)-t)]\Delta \end{array}$$

Similarly, the aggregate energy consumption generated by user device *u* over the time period *T* can be expressed as

(20) $$\begin{array}{c} \begin{array}{c} Energy_{u}^{total}=\lim_{T\to \infty }\frac{1}{T}\sum_{t=1}^{T}[\left(1-x_{u}\left(t\right)\right)Energy_{u}^{local}\left(t\right)+x_{u}\left(t\right)\left(Energy_{u}^{trans}\left(t\right)\right] \end{array} \end{array}$$

where $Energy_{u}^{local}\left(t\right)$ denotes the energy consumption from local computation, i.e., $Energy_{u}^{local}\left(t\right)=\min\left\{E_{u}^{local}\left(t\right),\kappa T^{max}\left(t\right)\Delta {\left(F_{u}^{local}\right)}^{3}\right\}$, and $Energy_{u}^{edge}\left(t\right)$ represents the energy consumption of user device *u* during transmission, i.e., $Energy_{u}^{edge}\left(t\right)=\min\left\{E_{u}^{edge}\left(t\right),p_{u}T^{max}\Delta \right\}$.

In the context of a multi-user, multi-MEC server scenario, the objective is to minimize the system utility function for user device *u*, which is the weighted sum of user delay and energy consumption across a time period *T*. Consequently, the following problem formulation is constructed:

(21) $$\begin{array}{c} \begin{array}{c} P:Cost_{u}^{total}=\alpha Time_{u}^{total}+\left(1-\alpha \right)Energy_{u}^{total} \end{array} \end{array}$$

(22) $$s.t.\sum_{u\in U}w_{u}\leq W$$

where α is a delay weighting parameter used to balance the trade-off between system delay and energy consumption. This parameter governs the relative importance of the two components in the overall system cost and should be selected based on the specific application scenario. For instance, in latency-sensitive applications such as augmented reality or real-time video processing, the parameter should be assigned a higher value to emphasize the time component. Conversely, in scenarios where energy efficiency is critical, such as IoT devices with limited battery capacity, the parameter should be set to a lower value to prioritize energy consumption minimization. *W* represents the total communication bandwidth allocated to the system. The constraint ensures that the aggregate bandwidth allocated to each user device does not exceed the system’s total communication bandwidth.

## 5. Algorithm Design

Problem P is inherently complex to solve due to the need to optimize both discrete computational offloading decisions and continuous communication bandwidth resource allocation. We recognize that once the offloading decisions are established, the communication resource allocation problem transforms into a convex function. Thus, our strategy is to decompose Problem P into two subproblems, computational offloading and resource allocation, which are addressed separately.

### 5.1. Resource Allocation

We focus on the allocation of communication bandwidth at time slot *t*. Tasks can only be uploaded to the edge node via the wireless channel if they have not been discarded in the transmission queue of the user device. Consequently, we formulate the following problem for the user tasks in the system awaiting transmission at time slot *t*:

(23) $$P1:\min_{w}\sum_{u=1}^{U}\frac{A_{u}}{w_{u}}$$

where *A_u_* is given by $A_{u}=\frac{\left(\alpha +p_{u}-\alpha p_{u}\right)d_{u}\left(t\right)\sum_{e\in E}y_{u,e}\left(t\right)x_{u}\left(t\right)}{log_{2}\left(1+\frac{p_{u}g_{u,S}}{N_{0}}\right)}$ is independent of $w_{u}$. Problem P1 is subject to the constraint (22). The derivation of $A_{u}$ is detailed in Appendix A.

By calculating the second derivative of problem P1 with respect to $w_{u}$, we obtain

(24) $$\frac{\partial^{2}w}{\partial^{2}w_{u}}=\frac{2A_{u}}{{\left(w_{u}\right)}^{3}}\geq 0$$

Formulation (23) confirms that the optimization problem P1 is a convex optimization problem [37]. Therefore, we further construct the Lagrangian function for this optimization problem [38]:

(25) $$L\left(w_{u},\mu \right)=\sum_{u=1}^{U}\frac{A_{u}}{w_{u}}+\sum_{u=1}^{U}\mu \left(w_{u}-W\right)$$

where μ is the Lagrange multiplier associated with the constraint, and μ>0.

Through applying the KKT conditions, we can derive the optimal value $w_{u}^{*}$ and μ that satisfy the following Equations [39]:

(26) $$\left\{\begin{array}{c} \frac{\partial L(w_{u},\mu )}{w_{u}}=0\\ W-\sum_{u=1}^{U}w_{u}=0 \end{array}\right.$$

By further derivation, we can obtain the optimal solution $w_{u}^{*}$ as follows:

(27) $$w_{u}^{*}=\frac{W\sqrt{A_{u}}}{\sum_{u=1}^{U}\sqrt{A_{u}}}$$

i.e., the optimal allocated bandwidth for user device *u* at time slot *t*.

### 5.2. Task Offloading

In the preceding section, we derive the optimal communication resource scheme at time slot *t* when the offloading policies are determined through mathematical formulations. This section is primarily concerned with determining the optimal offloading policies. We convert the task offloading policy solution within the optimization problem into a Markov decision process that operates over a finite number of time slots.

A standard Markov decision process [40] is typically defined as a quaternion (S,A,P,R), where *S* represents the set of states observed from the environmental system, *A* represents the set of actions that the agent can perform depending on the state of the environment, $P=P(s_{t+1}|s_{t},a)$ is the state transition probability function, and *R* is the reward that is obtained by the agent executing an action at state *s*. The specific details of each element in our Markov decision process problem model are as follows.

#### 5.2.1. State

At the beginning of time slot *t*, each user device in the system observes the following state information from the environment:

(28) $$\begin{array}{c} \begin{array}{c} S_{u}\left(t\right)=\left(d_{u}\left(t\right),Wait_{u}^{local}\left(t\right),Wait_{u}^{trans}\left(t\right),Wait_{u}^{edge}\left(t\right),Q\left(t\right)\right) \end{array} \end{array}$$

It includes task size $d_{u}\left(t\right)$, queue situation $Wait_{u}^{local}\left(t\right)$, $Wait_{u}^{trans}\left(t\right)$, $Wait_{u}^{edge}\left(t\right)$; and history edge nodes’ active queue information Q(t), where $Q\left(t\right)=\{Q_{e}\left(0\right),Q_{e}\left(1\right),\ldots ,Q_{e}\left(t-1\right),Q_{e}\left(t\right),\ldots |e\in E\}$.

#### 5.2.2. Action

Upon the arrival of a task during time slot *t*, user device *u*, acting as an agent, must decide whether to process the task locally or at an edge node. If the decision is to process the task at an edge node, it also needs to decide which edge node to select for processing. Thus, the action space is defined as

(29) $$A_{u}\left(t\right)=\left(x_{u}\left(t\right),y_{u}\left(t\right)\right)$$

where $y_{u}\left(t\right)=\left(y_{u,1}\left(t\right),y_{u,2}\left(t\right),\ldots ,y_{u,e}\left(t\right)\right),e\in E$.

#### 5.2.3. Reward

The goal of reinforcement learning is to maximize the long-term average reward of the system. We define the reward obtained by the user device agent as the negative of the weighted average sum of delay and energy consumption under the time period *T*, expressed as follows:

(30) $$R_{u}=-Cost_{u}^{total}$$

The specific algorithm is detailed in the subsequent subsection.

### 5.3. BPDRL Algorithm

**Figure 3:**
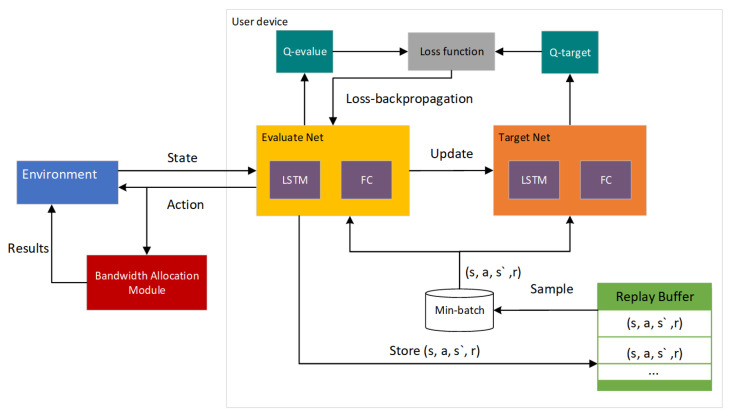
Algorithmic framework.

In this section, we propose a deep reinforcement learning-based approach to obtain task offloading policies and an optimal resource allocation scheme. Each user device observes the state from the environment at the inception of each time slot. Firstly, the user device anticipates the current active state of edge nodes predicated on historical active information of edge nodes. Secondly, the user device selects an appropriate action per the state and receives the corresponding bandwidth allocation. Subsequently, the tuples of state, action, and reward information, along with the historical active level information of edge nodes, are stored in an experience repository. Small batches of data are extracted from this experience repository, and the sampled data are then used to train the network. Through iterative learning, the network learns the mapping policy π from state–action pairs to Q-values to obtain the offloading strategy that maximizes the long-term cumulative rewards. The entire process is shown in Figure 3.

Our model is constructed on the architecture of deep Q-learning, an effective approach for addressing complex, high-dimensional problems characterized by long-term temporal dependencies. The deep Q-learning technique employs a deep neural network with initialized parameters to forecast the Q-values of state–action pairs. By leveraging mechanisms such as a replay buffer and a dedicated target network, the neural network is trained to refine its predictions of Q-values. By continuous iterative updating, the optimal policy $\pi^{*}$ is obtained. Its loss function is calculated as $L\left(\theta \right)=E[\left(R_{t}+\gamma \max Q\left(S_{t+1},A_{t+1}|\theta^{-}\right)-Q{\left(S_{t},A_{t}|\theta \right)}^{2}\right]$, where γ represents the discount factor for future rewards, θ represents the neural network parameters, which are periodically updated, and $\theta^{-}$ signifies the most current neural network parameters [41].

To tackle the challenge faced by user devices in not being aware of the dynamic load status of edge nodes, we integrate an LSTM prediction layer into the neural network. This enables user devices to anticipate the real-time state of edge nodes based on their historical load data, thus ensuring a potential load balance among edge nodes. Furthermore, following the task offloading decision, user devices acquire the optimal communication bandwidth allocation value according to the system conditions in the current time slot, ensuring maximum utilization of network resources. Algorithm 1 delineates the detailed procedure of our proposed dynamic computation offloading and resource allocation algorithm, BPDRL. The proposed BPDRL algorithm is a distributed approach, where each user device independently makes offloading decisions based on its local observations and historical information. This distributed nature ensures that the algorithm can scale efficiently with the number of user devices and edge nodes, without requiring a centralized controller to manage all decisions.

The algorithm initiates by initializing the parameters of the primary evaluation network and the target network. It ascertains the current active status of edge nodes via an LSTM prediction layer. Thereafter, based on the obtained current state *s*, it employs an ϵ−greedy strategy to select offloading actions. The user device opting to offload its task to an edge node broadcasts the task status information to other user devices within the network and concurrently receives task information from other user devices offloading their tasks to edge nodes. Based on formulation (26), the optimal communication bandwidth allocation scheme is established. Subsequently, the user device carries out the offloading action and interacts with the environment to garner the reward *r* and the subsequent state information $s^{\prime }$ under action *a*. The experience tuple $\left(s,a,r,s^{\prime }\right)$ is then stored into the experience replay buffer, and learning is conducted by randomly sampling from the experience replay memory. The target Q-value max Q(s,a,θ) is computed using the target network. The parameters of the primary evaluation network are updated in accordance with the loss function L(θ), and the parameters of the target network are periodically updated.

Our algorithm not only considers the computation offloading and resource allocation problems in the MEC scenarios but also proposes an adaptive task offloading and resources allocation scheme based on the unknown load status of edge nodes in the current dynamic scenario. In this scheme, each user device can fully utilize the computational resources of edge nodes and the system’s communication resources while maintaining system stability through load balancing on edge nodes.

**Algorithm 1** Algorithm of BPDRL.
 **Input: **Episode E, learning rate α, discount factor γ, batch sample size B **Output: **offloading policy $x_{u}\left(t\right)$, $y_{u}\left(t\right)$, optimal communication resource allocation strategy $w^{*}$1:Initialize the current evaluation network $Q\left(s,a\right),\theta^{-}$;2:Initialize the target network $Q(s^{\prime },a^{\prime }),\theta$;3:Empty the experience replay buffer M;4:**for** episode = 1,2,...,E **do**5:    Obtain initial state *s* from the environment;6:    **while** t=T  **do**7:         LSTM layer predicts the current activity of edge nodes $Q^{\prime }\left(t\right)$;8:         **if** user device’s transmission queue has a task waiting to be transmitted **then**9:             Broadcast the task information to the network;10:           Receive task information from other user devices obtained from the network;11:           Get the optimal bandwidth allocation $w^{*}$ according to the formulation (26);12:      **end if**13:      Use ϵ−greedy policy to select an action $a_{u}\left(t\right)$ according to the current state $s_{t}$;14:      Get reward $r_{u}\left(t\right)$ and next state $s_{t+1}$;15:      Store transition $\left(s_{t},r_{u}\left(t\right),a_{u}\left(t\right),s_{t+2}\right)$ into the experience replay buffer M;16:      **if** t mod L == 0 **then**17:          Sample a random mini-batch B from M;18:          Calculate the loss according to L(⊆);19:          Update current evaluation network parameters with $\theta^{-}\leftarrow \theta^{\prime }$;20:      **end if**21:      Regular update the target network parameters with $\theta^{-}\leftarrow \theta$;22:      t←t+1;23:   **end while**24:
**end for**



Divergent from traditional reinforcement learning methods, our approach encompasses personalized training networks $dnn_{u}$ for each user agent to make independent offloading decisions and engage with the environment. Moreover, we utilize the long-term weighted cost of the system as the optimization objective to gauge the QoS for users. Furthermore, to ensure the implementation of training networks on the user side, we incorporate deep compression techniques, which involve quantizing and pruning the parameters within the training network to reduce network size and expedite model convergence. Algorithm 2 outlines the specific steps of quantization and pruning.
**Algorithm 2** Pruning and parameters quantization.1:**if** pruning indicator == 1 **then**2:   Obtain updated weights for the evaluation network $\omega^{-}$;3:   Creates a mask *M* based on a given threshold value;4:   Obtain network parameters after pruning with $\omega^{-}\leftarrow \omega^{-}*M$;5:**end if**6:**if** quantization indicator == 1 **then**7:   Obtain updated weights for the evaluation network $\omega^{-}$;8:   Multiply weight parameters $\omega^{-}$ by the scaling factor ς;9:   Convert results to an 8-bit signed integer type;10:  Obtain network parameters after quantization with $\omega^{-}\leftarrow int8\left(\omega^{-}*\varsigma \right)$;11:**end if**

### 5.4. Complexity Analysis

The complexity of the BPDRL algorithm can be broken down into two main components: the training phase and the inference phase. We define *N* as the quantity of multiplication operations necessary for a single training iteration of the deep neural network, *E* as the total number of training episodes, and *B* as the batch size of samples utilized for training in each episode. Given that *K* tasks are stochastically generated by user device *u* over the time period *T*, the computational complexity of the proposed BPDRL algorithm during the training phase can be articulated as O(NEBK). During the inference phase, each user device uses the trained neural network to make offloading decisions in real time. The complexity of this phase is O(N) per time slot, as it involves a single forward pass through the neural network. It should be noted that the training and inference of neural networks introduce additional computational overhead. However, as this study focuses on optimizing the long-term average system cost, these additional computational costs are not explicitly considered in the analysis.

## 6. Experimental Results

Simulation experiments were carried out on a personal computer with a 3.20 GHz AMD Ryzen 7 5800H processor. PyCharm 2022.1.3 (Community Edition) served as the integrated development environment for implementing the experiments, and TensorFlow was utilized to construct the deep neural network model in Python.

In this section, we initially present the simulation experiment’s parameter settings and the benchmark methods for comparative analysis. Subsequently, we assess the convergence of the proposed algorithm and compare its performance with the baseline approaches under various scenarios, thereby highlighting the efficacy of our algorithm. Lastly, we discuss the influence of deep compression techniques on the proposed algorithm.

### 6.1. Parameter Settings

We simulate a circular area with a 50 m coverage radius, housing a base station equipped with multiple MEC nodes [10]. User devices are uniformly distributed across the area, engaging in wireless communication with the base station. The channel gain from user device *u* to the base station *S* is defined as $g_{u,S}=d^{-\theta }$, where *d* denotes the Euclidean distance between user device *u* and the base station *S*, and θ is the path loss factor [42]. Other specific parameter settings are detailed in Table 3.

To demonstrate the advantages of our proposed algorithm in maintaining system load balancing performance, we compare it with the following baseline algorithms:

(1) Random Offloading (Random): For arriving tasks, each user device randomly chooses an offloading strategy, either processing the task locally or uploading it to an edge node for MEC server processing. This scheme can achieve load balancing of edge nodes in a static environment.
(2) Deep Q-Network Offloading [26] (DQN): In a static communication environment, this algorithm considers the impact of latency and dynamic edge node loads on the system, employing a DQN-based approach to determine the offloading strategy.
(3) Hybrid Artificial Bee Colony Algorithm [44] (HABC): The algorithm employs a heuristic approach that integrates the strengths of genetic algorithms and simulated annealing to address the task offloading problem under constrained edge server resources.

### 6.2. Algorithm Convergence

**Figure 4:**
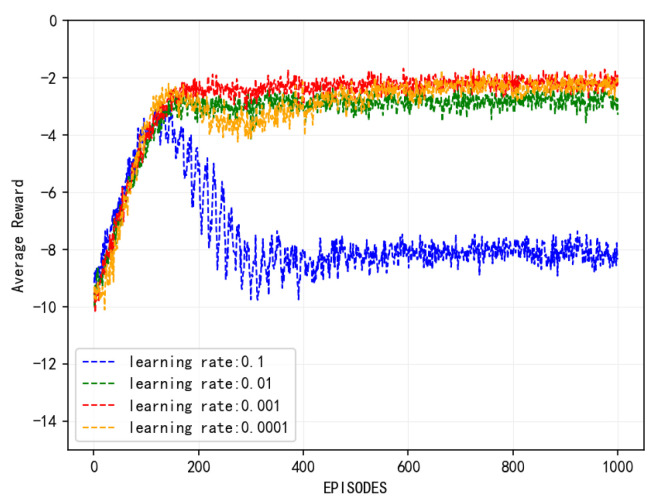
Convergence of algorithm at different learning rates.

Figure 4 illustrates the convergence behavior of the proposed algorithm under various learning rates. It is evident that the algorithm converges across all tested learning rates. Specifically, at a learning rate of 0.1, the algorithm exhibits the lowest convergence reward and the poorest performance. With a learning rate of 0.0001, the algorithm converges slowly, stabilizing at around 400 iterations. At a learning rate of 0.001, the algorithm converges more rapidly and achieves the peak reward value. The comparative results indicate that an excessively high learning rate can result in training oscillations, preventing the model from reaching the global optimal solution. Conversely, an overly low learning rate can lead to slow convergence and potential overfitting, thereby diminishing the model’s generalization capability. Therefore, selecting an appropriate and effective learning rate is crucial for algorithm performance.

In addition to the learning rate, the discount factor γ is another critical hyperparameter that directly influences the agent’s performance by determining the weight given to future rewards. We tested γ values in the range of 0.8 to 0.99. A smaller γ value (e.g., 0.8) biases the agent toward immediate rewards, leading to suboptimal solutions in tasks requiring long-term planning. Conversely, a larger γ value (e.g., 0.99) encourages the agent to prioritize long-term rewards but may result in slow convergence due to overemphasis on distant outcomes. Empirical results indicate that γ = 0.9 strikes a good balance, enabling efficient exploration of long-term strategies without sacrificing convergence speed. Based on these analyses, subsequent experiments were conducted using the hyperparameter settings of α = 0.001, γ = 0.9, a batch size of 32, and a replay buffer size of 500.

### 6.3. Algorithmic Comparison

In this segment, we assess the comparative performance of various algorithms with respect to system load balancing as the task arrival rate changes. Additionally, we evaluate the scalability of different algorithms under varying numbers of user devices. To quantify the load balancing efficacy in a dynamic MEC environment, we employ metrics such as the task drop rate, the average system cost, and the ratio of tasks processed locally or offloaded to edge nodes against the total number of tasks generated over a defined period. These metrics are widely recognized and utilized in numerous research studies [25,26,32].

#### 6.3.1. Impact of Varying Task Arrival Rates

**Figure 5:**
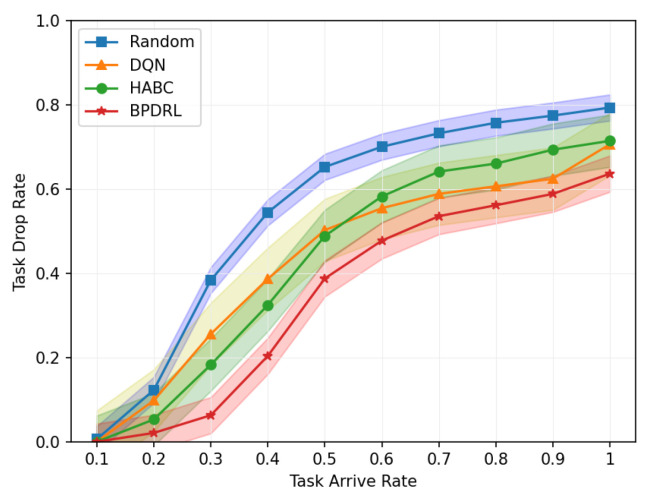
Comparison of task drop rates under different task arrival rates.

**Figure 6:**
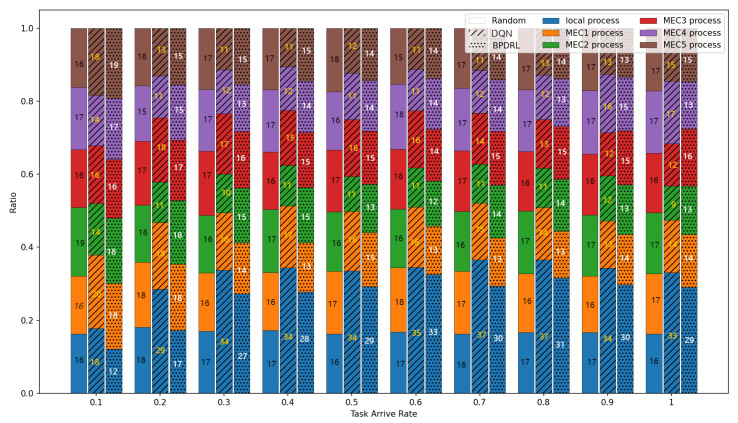
Comparison of proportion of tasks with different offloading methods under different task arrival rates.

Figure 5 delineates the comparison of task drop rate across varying task arrival rates. As the task arrival rate escalates, the task drop rates for all algorithms increase correspondingly. The proposed BPDRL algorithm effectively predicts the load levels of edge nodes in advance, ensuring optimal utilization of computational resources while avoiding excessive load. Consequently, it achieves the lowest task drop rate, even at a task arrival rate of 1. Notably, at a task arrival rate of 0.4, the BPDRL algorithm reduces the task drop rate by 47% compared to the DQN algorithm and by 37% compared to the HABC algorithm. These results highlight the superior robustness and stability of the BPDRL algorithm in high-load environments. Figure 6 presents the comparative distribution of tasks processed locally or offloaded to edge nodes as a percentage of the total tasks generated over a specified period under varying task arrival rates. It is evident that under the Random approach, user devices indiscriminately offload tasks to edge nodes. However, this strategy overlooks the system’s dynamic attributes, leading to elevated task drop rates. In contrast to the DQN algorithm, our BPDRL algorithm demonstrates a reduced proportion of tasks processed locally and a more equitable distribution of tasks offloaded to various edge nodes. This outcome can be credited to our algorithm’s implementation of a more efficient offloading strategy coupled with an optimal resource allocation scheme, which stabilizes loads across different edge nodes and ensures consistent system performance.

**Figure 7:**
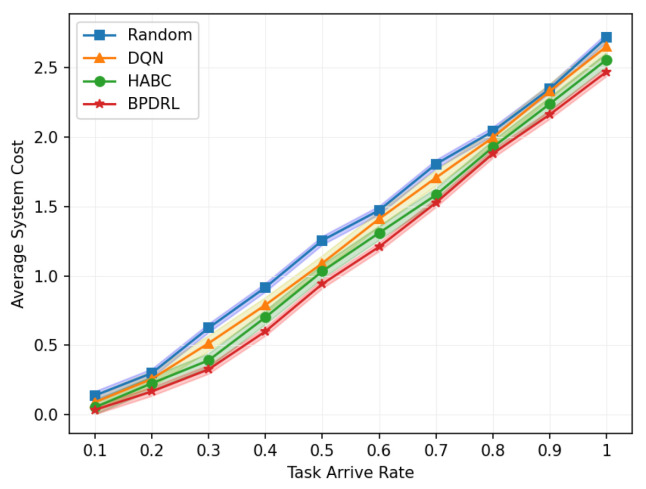
Comparison of average system cost under different task arrival rates.

Figure 7 illustrates the average system cost under different task arrival rates with the delay weighting parameter α set to 0.9. As the system’s task processing demands increase, the average system cost rises accordingly. The figure indicates that our proposed method consistently achieves the lowest average system cost compared to the other three algorithms. At a task arrival rate of 0.6, our proposed algorithm reduces the overall system cost by 14%, 18%, and 8% compared to the DQN, Random, and HABC algorithms, respectively. This reduction is due to our algorithm’s capability to dynamically devise offloading strategies based on the projected load status of the edge nodes and to adjust resource allocation in real time according to different offloading strategies employed by user devices.

**Figure 8:**
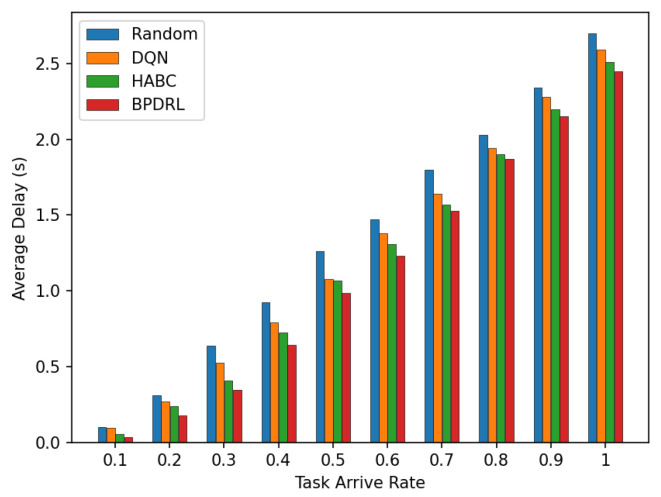
Comparison of average delay under different task arrival rates.

**Figure 9:**
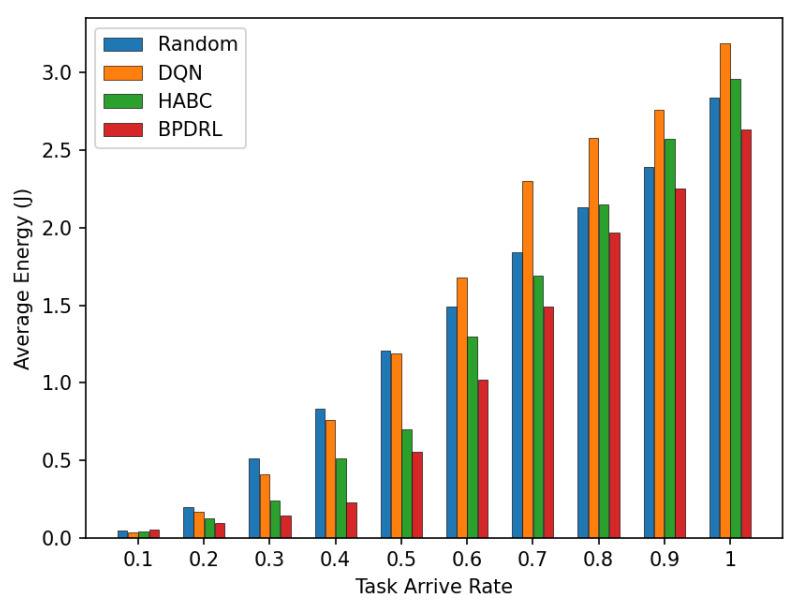
Comparison of average energy under different task arrival rates.

Figure 8 compares the average delay per time slot under various task arrival rates. Our approach consistently achieves the lowest average delay compared to the three baseline schemes. This advantage is attributed to our method’s priority selection to offload tasks to less burdened edge nodes for real-time tasks. Specifically, the incorporation of an LSTM-based prediction module into our algorithm enables user devices to predict the current load of each system node based on historical offloading data, thereby facilitating more accurate and efficient offloading decisions. Moreover, by optimally allocating real-time communication bandwidth to user devices, our approach enhances task transmission rates, leading to further improving the latency performance. Figure 9 illustrates the comparison of average energy consumption per time slot for different task arrival rates. As the task arrival rate increases, our proposed scheme consistently exhibits the lowest average energy consumption compared to the three baseline schemes. This superior performance is attributed to the ability of our scheme to maintain the lowest task drop rate across varying arrive rates by preferentially offloading tasks to edge nodes with greater available resources. This strategy minimizes energy wastage due to task drops and reduces the energy consumption of local devices.

#### 6.3.2. Impact of Varying User Device Populations

**Figure 10:**
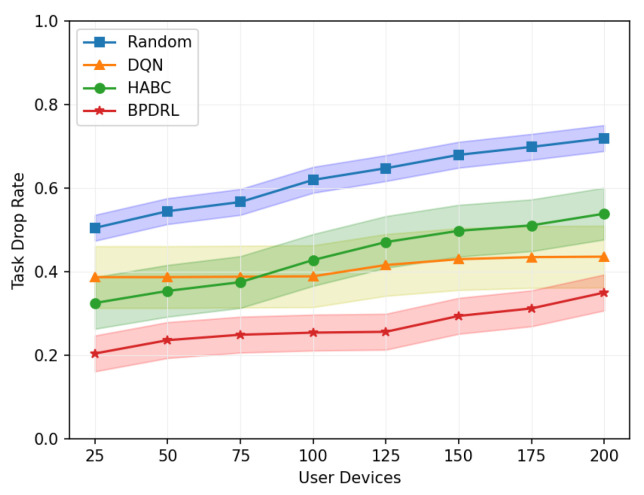
Comparison of task drop rates under different user devices.

**Figure 11:**
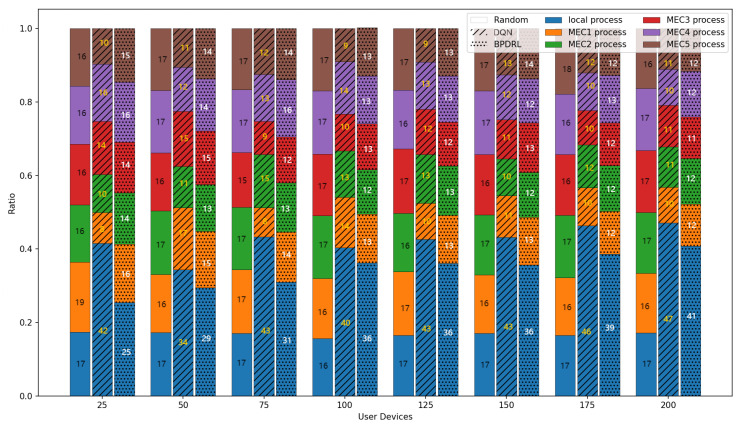
Comparison of proportion of tasks with different offloading methods under different user devices.

Figure 10 shows the comparison of the system task drop rate under different network scales defined by user device quantities. As the number of user devices increases, task drop rates for all schemes rise accordingly. However, our proposed approach consistently achieves the lowest task drop rates in scenarios with a large user base. This is due to the algorithm’s capability for each user to effectively predict real-time load dynamics at edge nodes, thereby fully utilizing the system’s communication and computing resources. This ensures dynamic load balancing and optimal resource utilization. When the number of user devices reaches 200, our approach reduces the task drop rate by 51.4%, 19.7%, and 35% compared to the Random, DQN, and HABC algorithms. Figure 11 compares the percentage of tasks processed locally or offloaded to the edge nodes relative to the total tasks generated over a given time period under different network scales. While the Random strategy distributes tasks evenly, it overlooks system dynamics, leading to higher task drop rates. In contrast, our approach dynamically maintains load balance among edge nodes based on real-time load levels, achieving a lower task drop rate. Compared with the DQN strategy, our approach tends to offload more tasks to the edge nodes and achieves a more balanced distribution of offloading decisions among the edge nodes, offering a significant advantage in addressing the load balancing challenges of edge nodes.

**Figure 12:**
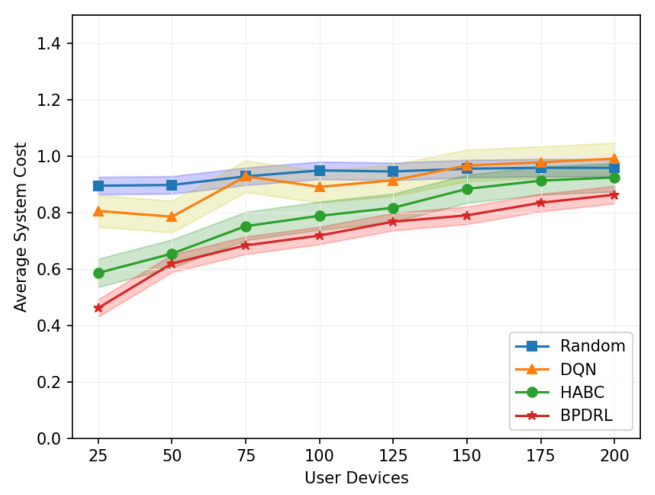
Comparison of average system cost under different user devices.

Figure 12 illustrates the variation in average system cost across different network sizes when the delay weighting parameter α is set to 0.7. The Random algorithm consistently maintains a high system cost, reflecting its suboptimal performance in resource utilization and task allocation. While the DQN algorithm achieves a lower system cost compared to the Random algorithm, its performance deteriorates significantly as the number of user devices increases, indicating limited adaptability to large-scale user networks. In contrast, the HABC algorithm demonstrates greater stability across varying network sizes, with only a slight increase in system cost as the number of user devices grows. However, the BPDRL algorithm consistently achieves the lowest system cost among all the baseline methods, with the most moderate growth trend as network size expands. These findings underscore the superior efficiency and robustness of the BPDRL algorithm in optimizing resource allocation and task offloading, effectively mitigating the impact of increased network scale on system performance.

**Figure 13:**
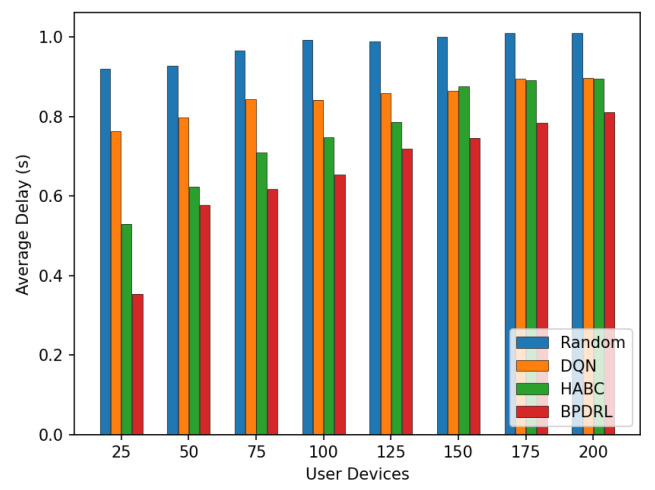
Comparison of average delay under different user devices.

**Figure 14:**
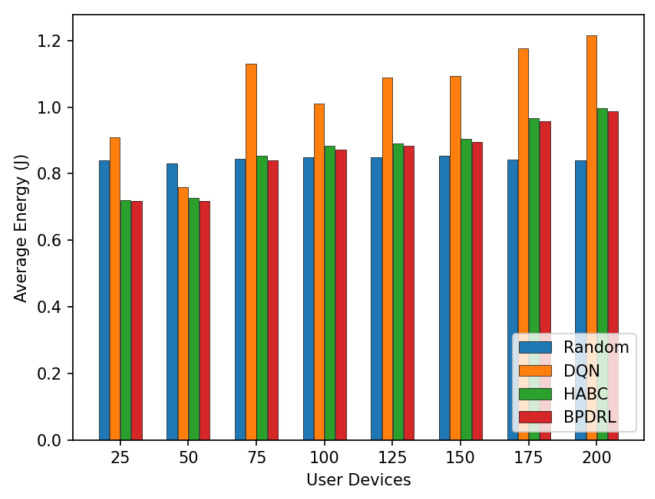
Comparison of average energy under different user devices.

Figure 13 compares the average delay per time slot under varying user device populations. As the number of user devices increases, the average delay rises for all schemes. Notably, the proposed method achieves the lowest average delay compared to the baseline schemes. This performance disparity arises because mobile edge nodes have limited computational resources. As the number of user devices grows, more tasks are offloaded to the edge nodes for processing. This surge in task volume often leads to overloaded edge nodes, causing a significant number of tasks to be dropped and thereby increasing the average delay. In contrast, the proposed algorithm enables each user device to predict the current load of edge nodes based on the system’s historical states. This predictive capability ensures that computational resources across edge nodes are utilized efficiently without causing overloads. As a result, tasks are processed more effectively within shorter time frames, significantly reducing the average delay. Figure 14 presents the average energy consumption per time slot under different network scales. With an increasing number of user devices, our scheme consistently maintains the lowest average energy consumption. When the number of user devices is 200, our method reduces the average system energy consumption by 18.8% and 1% compared to the DQN and HABC schemes.

#### 6.3.3. The Comparison of Runtime

To enhance the convergence performance of our proposed algorithm on user devices, we employ deep compression techniques to prune the model and quantize parameters. The model achieves pruning by removing parameters with smaller weights based on a predefined pruning threshold, thereby reducing the number of parameters and the associated computational complexity. Subsequently, an 8-bit quantization technique is employed to convert the pruned parameters from floating-point representations to low-precision 8-bit signed integers, effectively minimizing storage requirements while further reducing computational complexity.

**Figure 15:**
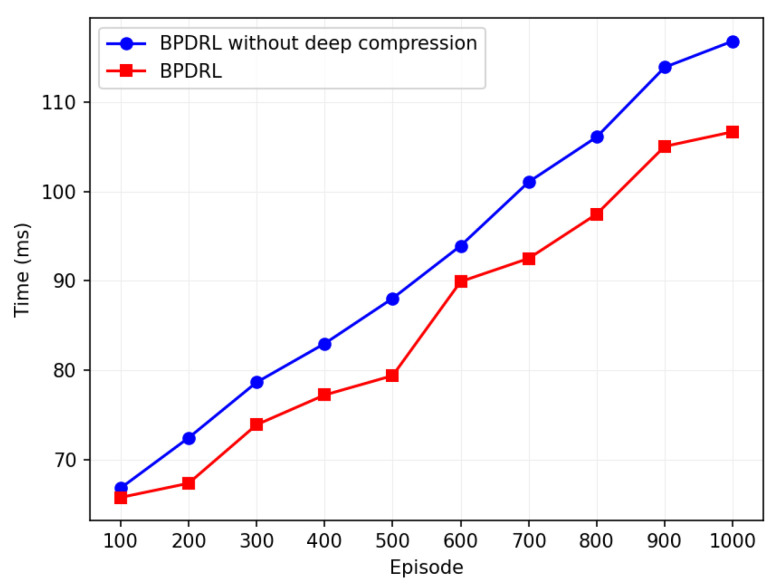
Comparison of whether to use deep compression.

Figure 15 compares the runtime of the algorithm with and without deep compression techniques. The horizontal axis represents training episodes, and the vertical axis indicates the average runtime per training episode every 100 episodes. The average runtime increases with training episodes, but the model with deep compression consistently shows lower runtime. Over 1000 training episodes, the algorithm with deep compression reduces runtime by 7.6% compared to the non-compressed version, with only a 3.5% reduction in task drop rate. The results demonstrate that the deep compression technique enhances the operational efficiency of the model while preserving its performance. Although pruning and quantization remove certain parameters, potentially impacting model accuracy, experimental verification reveals that the pruned and quantized model maintains a low task discard rate even under high task arrival rates. This highlights the model’s ability to sustain both accuracy and stability. Furthermore, as a 32-bit floating-point number requires 4 bytes of storage while an 8-bit integer requires only 1 byte, 8-bit quantization significantly reduces the model’s storage requirements and runtime memory footprint. This compression makes the model more suitable for deployment on resource-constrained devices. Thus, we deem the integration of deep compression techniques into the model to be beneficial.

### 6.4. Analysis of Statistical Significance

To evaluate the performance enhancements achieved by the BPDRL algorithm in task offloading and resource allocation within mobile edge computing environments, a comparative analysis was conducted against the HABC algorithm, DQN algorithm, and Random Offloading strategy. The evaluation focused on key performance metrics, including task drop rate and system latency. To ensure the reliability and consistency of the results, the experiment was repeated 10 times under identical conditions. A two-sample *t*-test was then performed to assess the statistical significance of the findings.

Table 4, Table 5 and Table 6 demonstrate that the BPDRL algorithm achieves a substantial improvement in task drop rate, with an average reduction of 37% compared to the HABC algorithm (*p*-value = 0.005). This result highlights the BPDRL algorithm’s enhanced ability to handle high-load tasks and mitigate task losses caused by insufficient resources. Furthermore, when compared to the DQN algorithm, the BPDRL algorithm achieves a 47% reduction in task drop rate (*p*-value = 0.000), further underscoring its superior performance in resource-constrained environments. Notably, the BPDRL algorithm reduces the task discard rate by 63% relative to the Random Offloading strategy (*p*-value = 0.000), a significant improvement that emphasizes its capability to make intelligent task offloading decisions in dynamic and complex environments. The BPDRL algorithm also demonstrates significant advantages in reducing system latency. Compared to the HABC algorithm, it achieves an average latency reduction of 9% (*p*-value = 0.010), indicating its ability to expedite task processing and thereby minimize user waiting times. Similarly, the BPDRL algorithm reduces system latency by 9% compared to the DQN algorithm (*p*-value = 0.008), further validating its effectiveness in optimizing task processing times. Moreover, the BPDRL algorithm achieves a substantial 20% reduction in system latency relative to the Random Offloading strategy (*p*-value = 0.000), highlighting its notable superiority in minimizing latency in dynamic and resource-constrained environments.

### 6.5. Real-World Deployment and Limitations of the BPDRL Algorithm

The implementation of the proposed algorithm involves several critical steps, including edge node deployment, user device integration, and system configuration. Specifically, edge nodes must be strategically deployed in proximity to user devices, such as base stations or routers, to minimize latency. Each edge node should be provisioned with adequate computational and storage resources to efficiently manage task offloading. User devices must be equipped with the requisite software to interface with the MEC system, enabling them to make offloading decisions based on the BPDRL algorithm. These devices should also be capable of monitoring their computational workload and communicating with the MEC system to receive bandwidth and computational resource allocations. The MEC system must be optimized to support the BPDRL algorithm, including the establishment of communication channels, resource allocation mechanisms, and the integration of the LSTM-based prediction layer. The system should be designed to accommodate dynamic variations in user workloads and task arrival rates, ensuring real-time adaptability and efficient resource utilization.

While the proposed BPDRL algorithm demonstrates feasibility in realistic MEC scenarios, several challenges persist when it is deployed under conditions of high user mobility and heterogeneous devices (HetDev). High user mobility results in frequent fluctuations in network conditions, particularly when users transition between different base stations or edge nodes. In such cases, efficient handover mechanisms are critical to ensuring seamless task offloading. Although the algorithm performs effectively in environments with stable communication link quality, it struggles to maintain low latency and high reliability in scenarios characterized by rapidly changing communication link conditions. In heterogeneous networks, user devices often possess varying computational capabilities, which can significantly impact their ability to process tasks locally or offload tasks to edge nodes. Ensuring that the algorithm operates seamlessly across devices with diverse hardware and software configurations poses an additional challenge. The experiments conducted in this study were performed within a homogeneous network setting, potentially limiting the algorithms’ compatibility and adaptability to heterogeneous network scenarios.

## 7. Conclusions

In this paper, we investigate the problem of task offloading and resource allocation in a multi-user multi-MEC system with the aim of minimizing the system long-term average cost. We propose a distributed scheme for offloading and resource allocation that takes into account queuing delays during task offloading. This strategy adeptly handles the dynamic and unpredictable load of edge nodes, optimizing the utilization of system resources. For tasks that newly arrive, user devices make adaptive offloading decisions to edge nodes based on the current system state, followed by the allocation of system bandwidth and computational resources. Simulation results demonstrate that our proposed approach markedly reduces the system’s task drop rate and long-term average cost while also sustaining commendable performance with respect to scalability across varying network sizes.

However, there are several limitations to our work. Firstly, the simulation experiments presuppose that users are stationary or exhibit low-speed mobility, thereby neglecting the impact of user mobility within the base station’s coverage area. This assumption may limit the applicability of our model to real-world scenarios where user mobility significantly affects task offloading and resource allocation decisions. Secondly, the task density parameter used in the simulations is set within a range of 20–51 cycles/bit, which may not fully reflect the task characteristics in modern computing systems, where tasks may exhibit much higher density values due to more complex computations or higher data requirements. This limitation could affect the generalizability of our results to real-world applications with different task profiles. Thirdly, our study does not consider inter-task dependencies, which are often present in practical scenarios. Additionally, data security and user privacy, which are crucial in MEC environments, are not explicitly addressed in this paper.

To address these limitations, future work will focus on integrating user mobility patterns and dynamic location changes into task offloading and resource allocation decisions. We will develop models and algorithms that incorporate inter-task dependencies to ensure more efficient and practical scheduling. Furthermore, future studies will investigate mechanisms to optimize task offloading and resource allocation while ensuring robust data security and user privacy, which are indispensable for the broader adoption of MEC systems. Additionally, we aim to refine the task density model to better reflect the requirements of modern computing systems.

## Figures and Tables

**Table 1:** Key features of the related research studies and our research.

Reference	Architecture		Algorithm	Research Issue		
	Distributed	Centralized		Offloading	Resource Allocation	Load Balancing
[11]	✓		ML-based	✓		✓
[12]	✓		DRL-based	✓		✓
[15]	✓		Greedy-based	✓		
[14]	✓		ML-based	✓		
[20]	✓		DRL-based	✓	✓	
[31]	✓		DRL-based	✓	✓	
Our paper	✓		DRL-based	✓	✓	✓

**Table 2:** Summary of key notations.

Notation	Definition
E	Edge nodes set
U	User devices set
T	Time slots set
$d_{u}\left(t\right)$	Task size generated by user device *u* at time slot *t*
$c_{u}\left(t\right)$	Task processing density generated by user device *u* at time slot *t*
$T^{max}$	Task maximum tolerable latency
$Task_{u}\left(t\right)$	The task generated by user device *u* at time slot *t*
$x_{u}\left(t\right)$	Task offloading decision
$y_{u,e}\left(t\right)$	Task offload policy: when a task chooses to offload to an edge node
$r_{u}\left(t\right)$	Uplink transmission rate of user device *u*
$w_{u}$	Bandwidth value assigned to user device *u* by the MEC system
$p_{u}$	Transmit power of user device *u*
$g_{u,S}$	Channel gain between user device *u* and base station *S*
$N_{0}$	Channel noise power
$F_{u}^{local}$	The local computing capability of user device *u*
κ	The effective capacitance factor
$f_{e}^{edge}\left(t\right)$	The computational resources allocated to edge node *e* in time slot *t*
$T_{u}^{trans}\left(t\right)$	Transmission delay of user device *u* in time slot *t*
$E_{u}^{trans}\left(t\right)$	Transmission energy of user device *u* in time slot *t*
$T_{u}^{local}\left(t\right)$	Local computational delay of user device *u* in time slot *t*
$E_{u}^{local}\left(t\right)$	Local computational energy of user device *u* in time slot *t*
$T_{u}^{edge}\left(t\right)$	Edge computational delay of user device *u* in time slot *t*
$Time_{u}^{local}\left(t\right)$	Time slot when a task is processed to completion in the local computational queue
$Time_{u}^{trans}\left(t\right)$	Time slot when a task completes transmission in the local transmission queue
$Time_{u}^{edge}\left(t\right)$	Time slot when a task is processed to completion on edge queue *u*
$Wait_{u}^{local}\left(t\right)$	The number of time slots a task has to wait before the local computational queue is processed
$Wait_{u}^{trans}\left(t\right)$	The number of time slots a task needs to wait before being transferred on the local transmission queue
$Wait_{u}^{edge}\left(t\right)$	The number of time slots a task is waiting to be processed on the edge queue *u*
$Q_{e}\left(t\right)$	Active queue, a set of queues on edge node *e* with tasks being processed or needing to be processed
$Cost_{u}^{total}$	The system utility function of the user device *u*, i.e., the weighted sum of user delay and energy consumption
$A_{u}$	The coefficient of the resource allocation problem that is independent of $w_{u}$

**Table 3:** Parameter settings.

Parameters	Value
Task size $d_{u}\left(t\right)$ [26,43]	[1–5] MB
Task processing density $c_{u}\left(t\right)$	[20–51] cycles/bit
Task arrive rate	[0.1–1]
Number of user devices	[25–200]
$F_{u}^{local}$	2 GHZ
$F_{e}^{edge}$	20 GHZ
*W*	100 MHZ
κ	$10^{-27}$
$p_{u}$	1 W
$N_{0}$	$10^{-13}$
$T^{max}$	3 s
Δ	0.1 s

**Table 4:** Significance analysis of BPDRL vs. HABC.

Metric	BPDRL	HABC	Mean Improvement	*p*-Value	*p* < 0.05
Task drop rate	0.204 ± 0.070	0.325 ± 0.097	−37%	0.005	Yes
Delay(s)	0.811 ± 0.051	0.895 ± 0.077	−9%	0.010	Yes

**Table 5:** Significance analysis of BPDRL vs. DQN.

Metric	BPDRL	DQN	Mean Improvement	*p*-Value	*p* < 0.05
Task drop rate	0.204 ± 0.070	0.387 ± 0.116	−47%	0.000	Yes
Delay(s)	0.811 ± 0.051	0.896 ± 0.091	−9%	0.008	Yes

**Table 6:** Significance analysis of BPDRL vs. Random.

Metric	BPDRL	Random	Mean Improvement	*p*-Value	*p* < 0.05
Task drop rate	0.204 ± 0.070	0.545 ± 0.051	−63%	0.000	Yes
Delay(s)	0.811 ± 0.051	1.010 ± 0.046	−20%	0.000	Yes

## Data Availability

Data are contained within the article.

## Appendix A

Once the offloading decision is determined, Equation (21) simplifies to a convex function that depends solely on the bandwidth $w_{u}$ allocated to user device *u*. The terms $\left(1-x_{u}\left(t\right)\right)\left(Time_{u}^{local}\left(t\right)-t\right)$ and $\left(1-x_{u}\left(t\right)\right)Energy_{u}^{local}\left(t\right)$ are independent of $w_{u}$. Therefore, they can be represented by the constants $C_{1}$ and $C_{2}$, respectively.

Given that we focus on bandwidth allocation for user devices with tasks that are not discarded within time slot *t*, the discarded tasks do not occupy bandwidth or computational resources. Consequently, only the tasks remaining in the transmission queue of user device *u* during time slot *t* need to be considered for communication bandwidth and edge computational resource allocation. Thus, Equation (21) can be further simplified as follows:

(A1) $$\begin{array}{c} \begin{array}{c} Cost_{u}^{total}=\lim_{T\to \infty }\frac{1}{T}\sum_{t=1}^{T}[\alpha x_{u}\left(t\right)\frac{d_{u}\left(t\right)\sum_{e\in E}y_{u,e}\left(t\right)}{w_{u}\log_{2}\left(1+\frac{p_{n}g_{u,S}}{N_{0}}\right)}+\\ \left(1-\alpha \right)x_{u}\left(t\right)P_{n}\frac{d_{u}\left(t\right)\sum_{e\in E}y_{u,e}\left(t\right)}{w_{u}\log_{2}\left(1+\frac{p_{n}g_{u,S}}{N_{0}}\right)}] \end{array} \end{array}$$

We consider real-time resource allocation within time slot *t*, which is derived by processing Equation (30) as follows:

(A2) $$\begin{array}{c} \begin{array}{c} Cost_{u}^{total}\left(t\right)=\frac{1}{w_{u}}\frac{\left(\alpha +p_{u}-\alpha p_{u}\right)d_{u}\left(t\right)\sum_{e\in E}y_{u,e}\left(t\right)x_{u}\left(t\right)}{log_{2}\left(1+\frac{p_{u}g_{u,S}}{N_{0}}\right)} \end{array} \end{array}$$

For simplicity in representation, we define $A_{u}=\frac{\left(\alpha +p_{u}-\alpha p_{u}\right)d_{u}\left(t\right)\sum_{e\in E}y_{u,e}\left(t\right)x_{u}\left(t\right)}{log_{2}\left(1+\frac{p_{u}g_{u,S}}{N_{0}}\right)}$ to represent the coefficients in Equation (30) that are independent of $w_{u}$.
